# Administration of Amyloid Precursor Protein Gene Deleted Mouse ESC-Derived Thymic Epithelial Progenitors Attenuates Alzheimer's Pathology

**DOI:** 10.3389/fimmu.2020.01781

**Published:** 2020-08-11

**Authors:** Jin Zhao, Min Su, Yujun Lin, Haiyan Liu, Zhixu He, Laijun Lai

**Affiliations:** ^1^Guizhou Provincial Key Laboratory for Regenerative Medicine, Tissue Engineering and Stem Cell Research Center, Department of Immunology, School of Basic Medical Sciences, Guizhou Medical University, Guiyang, China; ^2^Key Laboratory of Adult Stem Cell Translational Research, Chinese Academy of Medical Sciences, Guiyang, China; ^3^Department of Allied Health Sciences, University of Connecticut, Storrs, CT, United States; ^4^Department of Pediatrics, Affiliated Hospital of Zunyi Medical University, Zunyi, China; ^5^University of Connecticut Stem Cell Institute, University of Connecticut, Storrs, CT, United States

**Keywords:** Alzheimer's disease, amyloid-beta, thymic epithelial cells, embryonic stem cells, amyloid precursor protein, T cells

## Abstract

Alzheimer's disease (AD) is a devastating neurodegenerative disorder and the most common cause of dementia in older adults. Although amyloid-beta (Aβ) plaque deposition and chronic neuroinflammation in the central nervous system (CNS) contribute to AD pathology, neither Aβ plaque removal nor anti-inflammatory therapy has shown much clinical success, suggesting that the combinational therapies for the disease-causative factors may be needed for amelioration. Recent data also suggest that systemic immunity in AD should be boosted, rather than suppressed, to drive an immune-dependent cascade needed for Aβ clearance and brain repair. Thymic epithelial cells (TECs) not only play a critical role in supporting T cell development but also mediate the deletion of autoreactive T cells by expressing autoantigens. We have reported that embryonic stem cells (ESCs) can be selectively induced to differentiate into thymic epithelial progenitors (TEPs) *in vitro* that further develop into TECs *in vivo* to support T cell development. We show here that transplantation of mouse ESC (mESC)-TEPs into AD mice reduced cerebral Aβ plaque load and improved cognitive performance, in correlation with an increased number of T cells, enhanced choroid plexus (CP) gateway activity, and increased number of macrophages in the brain. Furthermore, transplantation of the amyloid precursor protein (APP) gene deleted mESC-TEPs (APP^−/−^) results in more effective reduction of AD pathology as compared to wild-type (APP^+/+^) mESC-TEPs. This is associated with the generation of Aβ-specific T cells, which leads to an increase of anti-Aβ antibody (Ab)-producing B cells in the spleen and enhanced levels of anti-Aβ antibodies in the serum, as well as an increase of Aβ phagocytosing macrophages in the CNS. Our results suggest that transplantation of APP^−/−^ human ESC- or induced pluripotent stem cell (iPSC)-derived TEPs may provide a new tool to mitigate AD in patients.

## Introduction

Alzheimer's disease (AD) is a devastating age-related neurodegenerative disorder, affecting over 34 million people worldwide (1). AD is characterized by progressive loss of memory and cognitive functions (1–5). The cognitive decline in AD is associated with hallmark protein aggregates, amyloid-beta (Aβ) plaques and neurofibrillary tangles, which are accompanied by neuroinflammation, and synaptic and neuronal loss (1–5). Aβ plaques play a central role in the pathogenesis of AD and are generated from proteolytic cleavage of amyloid precursor protein (APP) (6, 7). Aβ can accelerate neuronal cell death and neuronal tangle formation, affect synaptic function adversely and eventually cause neuron loss (2–4, 8). The accumulated Aβ plaques and neuroinflammation have led to numerous attempts over the years to treat AD, either by removing the Aβ plaques, or by systemic anti-inflammatory drug administration to arrest brain inflammation. However, the drugs tested thus far for AD have largely failed in the clinic (4, 9–12). These failures suggest that, although removal of Aβ plaques may be important, this approach alone is not enough to arrest or reverse cognitive loss. Furthermore, recent data also suggest that mitigating neuroinflammation in AD necessitates stimulation, rather than suppression of the immune system, to drive an immune-dependent cascade needed for the Aβ clearance and brain repair (4, 13, 14). It has been shown that anti-inflammation is an active mechanism mediated by recruitment of circulating immune cells to sites of brain pathology (15–17). In addition, systemic immune deficiency is associated with cognitive dysfunction (18) and accelerated AD pathology (19, 20).

T cells are the major component of the immune system. Multiple lines of evidence have suggested that T cells play an important role in the CNS maintenance and repair. For example, systemic T cell deficiency is associated with increased neuronal loss in animal models of CNS injury or AD (14, 19, 21). Systemic T cells not only participate in CNS repair, but are also needed for life-long brain plasticity (18, 22, 23). Both T cells and monocyte-derived macrophages recognizing brain antigens are required for coping with and helping heal brain damage during central nervous system (CNS) injuries (24–29). T cells present in the periphery play an important role in adaptive–innate immunity cross-talk and help in CNS repair (16, 19, 20). Furthermore, it has been suggested that autoreactive T cells that recognize CNS-specific antigens augment the recruitment of monocyte-derived macrophages to the brain (14, 26).

The thymus is the primary organ for T cell generation. It, however, undergoes age-dependent thymic involution, resulting in decreased numbers of T cells in the elderly. This reduction has direct etiological linkages with many diseases (30–34), including acceleration of the development and progression of AD (19). T cell development in the thymus depends on the thymic microenvironment, in which thymic epithelial cells (TECs) are the major component (35–41). However, TECs undergo both qualitative and quantitative loss over time, which is believed to be the major factor responsible for age-dependent thymic involution (30–34). It is well-known that embryonic stem cells (ESCs) have the dual ability to propagate indefinitely *in vitro* in an undifferentiated state and to differentiate into many types of cells (42). We have reported that ESCs can be selectively induced to generate TEPs *in vitro* (43–46). When transplanted into young or old mice, the ESC-TEPs further develop into TECs, reconstitute the normal thymic architecture, and promote T cell generation, resulting in increased number of functional T cell in the periphery (43–46).

We hypothesized that AD aged mice and patients have a very severe defect in the thymic microenvironment and that transplantation of ESC-TEPs into AD mice would rejuvenate the aged thymic microenvironment, leading to an increased number of functional T cell in the periphery, resulting in attenuated AD pathology. It is well-known that TECs, especially medullary TECs (mTECs), are involved in the deletion of autoreactive T cells. We have demonstrated that transplantation of ESC-TEPs expressing disease-causative self-antigen results in the deletion of the antigen-specific autoreactive T cells (47, 48). Our hypothesis further proposes that transplantation of APP gene-deleted ESC-TEPs would lead to the generation of Aβ-specific autoreactive T cells that could help the production of other Aβ-specific immune cells to clear the Aβ plaques in the CNS.

We show here that transplantation of APP gene deleted (APP^−/−^) or their wild-type (APP^+/+^) mouse ESC (mESCs)-derived-TEPs results in enhanced thymopoiesis, increased T cell number, especially IFN-γ-producing cells, in the periphery, enhanced choroid plexus (CP) gateway activity, and enhanced recruitment of macrophages into the brain. Consequently, these mice have reduced Aβ deposits in the brain and improved cognitive performance. Furthermore, transplantation of APP^−/−^ mESC-TEPs has a greater effect than that of APP^+/+^ mESC-TEPs in clearance of Aβ deposits in the CNS and reversal of cognitive decline. This is related to the generation of Aβ-specific T cells, increased numbers of anti-Aβ antibody (Ab)-producing B cells in the spleen, increased levels of anti-Aβ Ab in the serum, and enhanced function of macrophages to phagocytose Aβ in the brain. Our results suggest that human ESC (hESC)- or induced pluripotent stem cell (iPSC)-derived TEPs, especially APP^−/−^ hESC or iPSC-TEPs, may serve as a novel tool to modify AD pathology.

## Materials and Methods

### Mice

3xTg-AD, APP/PS1, C57BL/6 (B6) mice were purchased from Jackson Laboratory. The mice were used in accordance with a protocol approved by the Institutional Animal Care and Use Committee of the University of Connecticut.

### Cell Culture

B6 mESC line (from Cyagen, Santa Clara, CA) were cultured in ESGRO Complete Plus Serum-free Clonal Grade Medium with GSK3β inhibitor supplement (Millipore, Temecula, CA). For TEP differentiation, mESCs were first induced to differentiate into definitive endoderm, and then TEPs in the presence of BMP-4, FGF 7, FGF10, and EGF, as well as rFOXN1 and rHOXA3 protein as we previously described (43).

### Genome Editing

The APP gene in mESCs was knocked out by the Clustered Regularly Interspaced Short Palindromic Repeats (CRISPR) and CRISPR-associated protein (Cas9) genome editing. B6 mESCs were transfected with APP-specific double nickase plasmids or control double nickase plasmids (from Santa Cruz Biotechnology). The cells were screened to obtain APP^−/−^ and APP^+/+^ mESCs. The information of the plasmids and gRNA sequences are shown in Supplemental Figure 1.

### Intrathymic Injection

Mice were anesthetized and injected with 5 × 10^4^ cells in 10–20 μl PBS into the thymus posterior to the upper sternum using a 26–28 gauge needle as described (49).

### Reverse Transcription Polymerase Chain Reaction (RT-PCR) and Real-Time Qualitative RT-PCR (qRT- PCR)

Total RNA was extracted from tissues or cells using a Nucleo Spin RNA II kit (Macherey-Nagel, Düren, Germany). The RNA was converted into complementary DNA using High Capacity cDNA Reverse Transcription Kit (Invitrogen, USA). RT-PCR was performed with GoTaq® Green Master Mix (Promega, USA). qRT- PCR was performed with the Power SYBR green master mix (Applied Biosystems, UK) using the 7500 real-time PCR system (Applied Biosystems, UK). The primer sequences are shown in Supplemental Table 1.

### Western Blot Analysis

GFP^+^ mESC-TECs were purified from the thymocytes using a magnetic-activated cell sorter immunomagnetic separation system (Mitenyi Biotec). The cells were collected and lysed. Equal amounts of denatured proteins were loaded onto a 4–12% Bis-Tris gel (Invitrogen, Carlsbad, CA), electrophoresed and transferred onto a PVDF membrane (Invitrogen). The membranes were blocked with 5% nonfat milk in TBST (mixture of Tris-Buffered Saline and Tween 20), and incubated with anti-mouse APP monoclonal antibody (Invitrogen) at 4 degree overnight. The membranes were then incubated with goat anti-mouse IgG HRP-conjugated secondary antibody and developed with a SuperSignal West Pico chemiluminescence substrate (Thermo Scientific, Rockford, IL).

### Immunohistochemistry

The brain tissues were incubated in a fixative solution, embedded in OCT medium, snap frozen, and subsequently cut into 6 μm sections. The cultured cells were incubated with primary antibodies. The following primary antibodies were used: mouse anti-Aβ (clone 6E10,) and rabbit anti-GFAP (Biolegend, USA). After washing, the sections were incubated with fluorochrome-conjugated secondary antibody, counterstained with 4′, 6′-diamidino-2-phenylindole (DAPI) and observed under a Nikon A1R Spectral Confocal microscope (Nikon, Kanagawa, Japan). To quantify the staining intensity, total cells and background fluorescence intensity were measured using ImageJ software (NIH, USA), and the intensity of specific staining was calculated as described (4).

### Flow Cytometry Analysis

To analyze TECs, the thymi were incubated at 37°C in 0.01 (w/v) liberase (Roche, Nutley NJ) and 0.02% (w/v) DNAse I (Roche) with regular and gentle agitation as described (50). A single-cell suspension of tissues was stained with fluorochrome-conjugated antibodies directly or indirectly as described (51). For intracellular staining, the cells were first permeabilized with a BD Cytofix/Cytoperm solution for 20 min at 4°C. The following antibodies were used: CD4, CD8, EpCAM1, CD45, CD11b, F4/80, IFNγ, Ly6c, and SRA1 (BioLegend, San Diego, CA, or ThermoFisher Scientific), Keratin (K) 5 (Covance, Dallas, TX), and K8 (US Biological, Salem, MA). The samples were analyzed on an LSRFortessa X-20 Cell Analyzer (BD Biosciences). Data analysis was performed using FlowJo software (Ashland, OR).

### T Cell Proliferation Assay

Splenocytes were stained with 5 μM CFSE (ThermoFisher Scientific) for 15 min. at 37°C. The cells were then cultured in a 96-well flat-bottom plate in the presence of plate-bound Aβ40 or Aβ42 (Anaspec, USA) and anti-CD3 antibody for 3 days. The cells were then stained with anti-PE labeled-CD4 and APC labeled-CD8 antibodies and analyzed for CFSE levels by T cells using flow cytometry.

### ELISA Assay for Anti-Aβ40 or Anti-Aβ42 Antibody

Aβ40 or Aβ42 (Anaspec, USA) was coated on 96-well microplates overnight at 4°C, then blocked with blocking buffer (2% BSA+5% goat serum in PBS) for 2 h at room temperature. The serum samples diluted into 1:1000 were added to the plates and incubated 2 h at room temperature. After washing, HRP-conjugated goat anti-mouse IgG (Biolegend) was added to the plates and incubated for 1 h. The reaction was developed by TMB substrate (Thermo Scientific, USA) and stopped with 0.1 N HCl. The microplate was read at 450 nm under a microplate reader (Bio-Tek, ELX800, USA). The antibody concentrations were calculated using a standard curve generated with known concentrations of anti-Aβ antibody.

### Soluble Aβ Protein (sAβ) Isolation and Quantification

Brain parenchyma was dissected, snap-frozen and kept at −75°C until homogenization. The samples were homogenized, and the supernatants were collected and detected for the concentrations of Aβ_1−40_ and Aβ_1−42_ by ELISA as described (13).

### B Cell ELISpot Assay

MultiScreen-IP plates (Millipore, Billerica, MA) were washed with 70% ethanol, rinsed three times with PBS, coated with Aβ40 (4 μg/ml) or Aβ42 (4 μg/ml) at 4°C overnight. The plates were blocked with blocking buffer (2% BSA in RPMI medium). 1 × 10^4^ splenocytes were added into the plates and incubated for 48 h. The plates were washed 6 times with 0.25% Tween 20 (Sigma, USA) in PBS, incubated with HRP-conjugated goat anti-mouse IgG (H+L) (Biolegend) for 1 h, developed with a DAB Peroxidase Substrate Kit (Vector, USA), and counted for ELISpots (52).

### Amyloid Phagocytosis Assay

HiLyte Fluor 647 Beta-Amyloid (1–42) (Anaspec) was resuspended in Tris/EDTA (pH 8.2) at 20 mM and then incubated in the dark for 3 days at 37°C to promote aggregation. Macrophages in suspension were pretreated in low serum medium as described (53). The HiLyte Fluor 647 Beta-Amyloid was added and incubated for 5 h. Cells were stained with macrophage markers; amyloid phagocytosis by the macrophages was determined by flow cytometry (53).

### Barnes Maze

Barnes Maze was conducted as previously described (54, 55). Briefly, each mouse was placed in the center of the maze and subjected to aversive stimuli. Mice were trained 4 training trials per day for 5 days, and a probe test was performed 24 h after the last training trial. The latency and number of errors were recorded for the training tail and probe test.

### Novel Object Recognition (NOR) Test

A NOR test was conducted as previously described (54–56). Briefly, mice were trained by allowing them to explore two identical objects placed at opposite ends of the arena for 10 min. 24 h later, mice were tested with one copy of the familiar object and one novel object of similar dimensions for 3 min. The time spent on exploring and sniffing of each object was recorded. The NOR index represents the percentage of time mice spent exploring the novel object.

### Statistical Analysis

*P*-values were based on the two-sided Student's *T*-test. A confidence level above 95% (*p* < 0.05) was determined to be significant.

## Results

1. Deletion of the APP gene in mESCs to generate APP^−/−^ mESCs and generation of TEPs from APP^−/−^ and APP^+/+^ mESCs *in vitro*.

**Figure 1:**
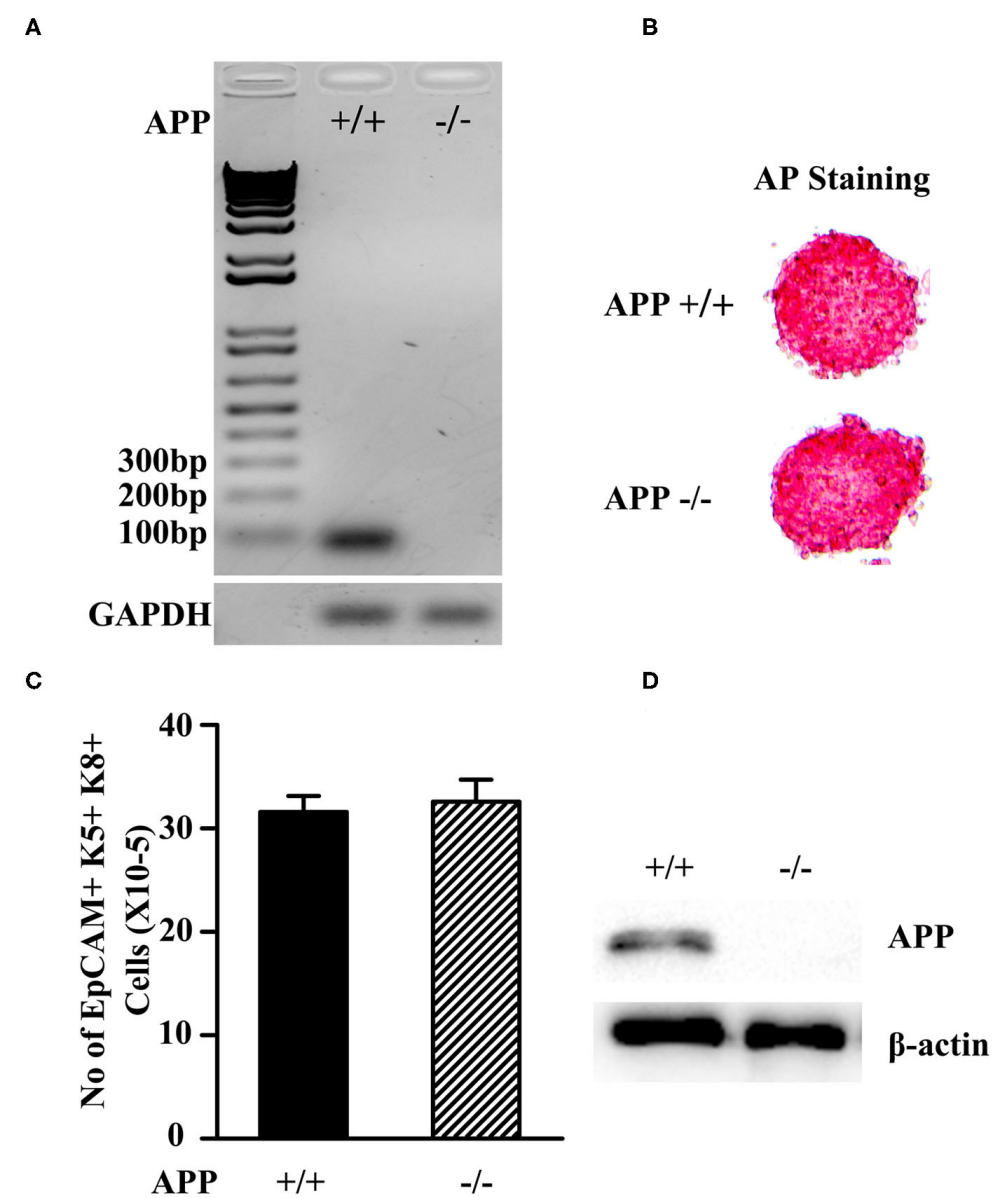
Characterization of APP^−/−^ and APP^+/+^ mESCs. **(A)** The expression of the APP mRNA in APP^−/−^and APP^+/+^ mESCs was measured by RT-PCR. **(B)** APP^−/−^ and APP^+/+^ mESCs were assessed for the expression of the pluripotent marker AP. **(C)** APP^−/−^ and APP^+/+^ mESCs were induced to differentiate into TEPs *in vitro*. The number of mESC-derived TEPs (EpCAM1^+^K5^+^K8^+^) were analyzed by flow cytometry. **(D)** APP^−/−^ and APP^+/+^ mESC-EpCAM1^+^ TEPs were injected into the thymus of syngeneic C57BL/6 mice. Two months later, GFP^+^ mESC-TECs were purified from the thymus, and analyzed for the expression of APP protein by Western blot. The data are presented from 3 independent experiments.

Since Aβ is produced from proteolytic cleavage of APP, we deleted the APP gene in mESCs using CRISPR and Cas9 genome editing. B6 mESCs were transfected with APP-specific double nickase plasmids that contain the APP-specific-guide RNAs, and the Cas9 nuclease and GFP genes. The cells were screened in puromycin to obtain APP^−/−^ mESCs. The gene deletion was confirmed by RT-PCR with one of primers spanning the gRNA region (Figure 1A). The cells that were transfected with control double nickase plasmids containing non-targeting scrambled gRNA, Cas9, and GFP genes were used as a control (APP^+/+^ mESCs).

Both APP^−/−^ and APP ^+/+^ mESCs were positive for alkaline phosphatase (AP) activity, indicating that the mESCs were in an undifferentiated state (Figure 1B). We then induced the APP ^−/−^ and APP^+/+^ mESCs to differentiate into TEPs *in vitro* following our protocol (43). After the differentiation, both APP^−/−^ and APP^+/+^ mESC-derived cells contained comparable numbers of EpCAM1 positive cells that co-expressed K5 and K8, a phenotype of TEPs (Figure 1C). We purified EpCAM1^+^ TEPs from APP^−/−^ and APP^+/+^ mESC-derived cells and injected an equal number of the TEPs into the thymus of syngeneic mice. Two months later, the mESC-TEPs generated comparable numbers of GFP^+^ mESC-derived TECs that accounted for 51–58% of total TECs.

Western blot analysis showed that purified GFP^+^ APP^+/+^ mESC-TECs expressed APP protein, whereas GFP^+^ APP^−/−^ mESC-TECs did not (Figure 1D and Supplemental Figure 2). Together, these results indicate that the APP gene has been deleted in the APP^−/−^ mESCs, and that the deletion does not affect the differentiation ability of mESCs into TEPs and TECs.

2. Both APP^−/−^ and APP^+/+^ mESC-TEP-transplanted AD mice have an improved cognitive performance, and APP^−/−^ mESC-TEP-transplanted mice perform better than APP^+/+^ mESC-TEP-transplanted mice.

**Figure 2:**
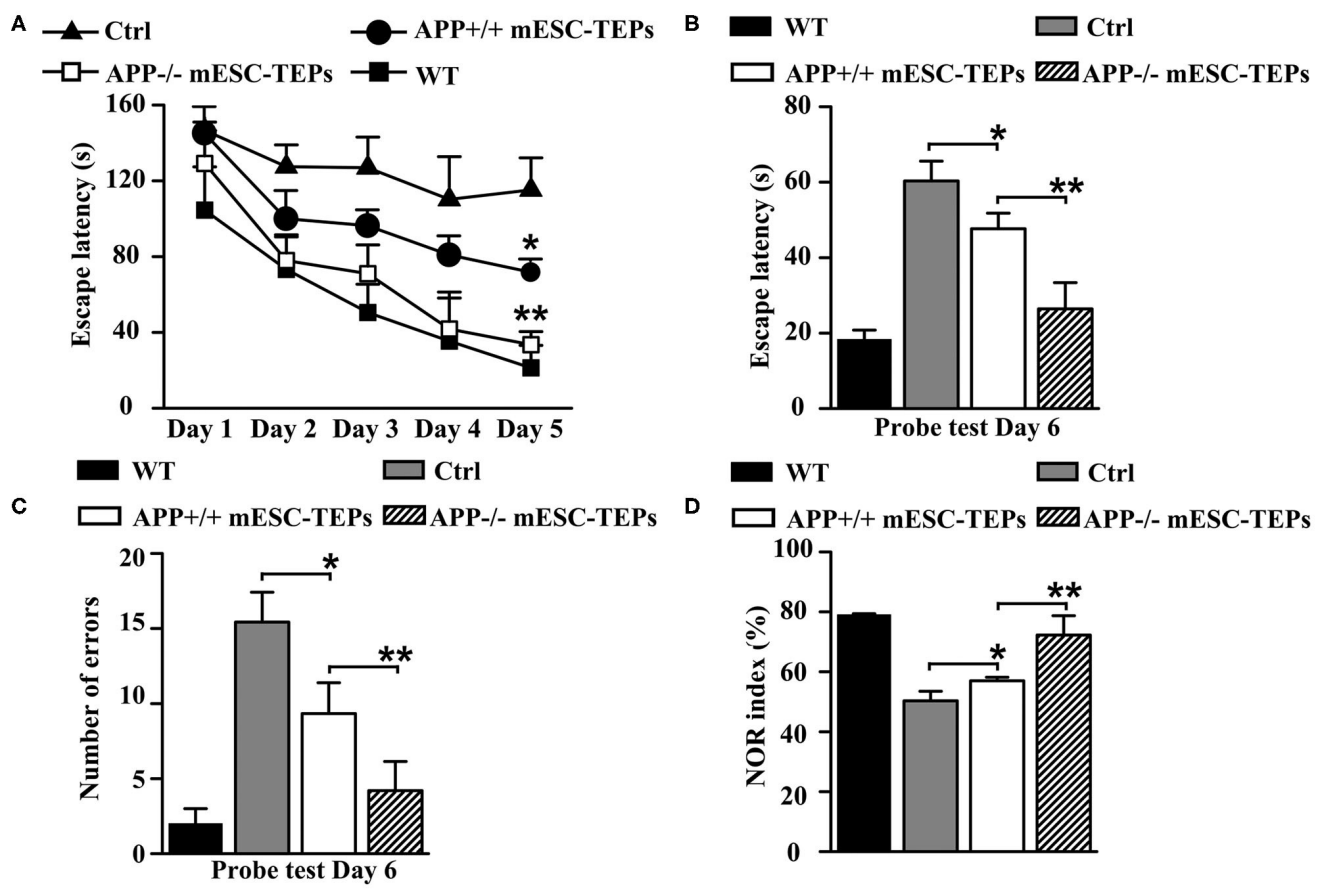
Transplantation of APP^−/−^ and APP^+/+^ mESC-TEPs improves cognitive performance in AD mice and APP^−/−^ mESC-TEPs are more effective than APP^+/+^ mESC-TEPs. **(A–D)** 3XTg-AD mice (12-month-old) were injected i.t. with APP^−/−^ EpCAM1^+^ mESC-TEPs, APP^+/+^ EpCAM1^+^ mESC-TEPs, or control cells (5 × 10^4^ cells/mouse). Age-matched untreated WT mice were also used as controls. Two months later, the mice were evaluated for cognitive performance by Barnes Maze and Object Recognition tests. **(A)** The escape latency during the training period, and **(B,C)** the escape latency and the number of errors committed during the probe trial are shown. **(D)** NOR index was determined as the time spent interacting with the novel object divided by the total time of exploration during the testing phase. The data are expressed as mean ± SD from one of three independent experiments with similar results (four to eight mice per group in each experiment). *p < 0.05 vs. control cell group, **p < 0.05 vs. APP^+/+^ mESC-TEP group.

To determine whether transplantation of APP^−/−^ and APP^+/+^ mESC-TEPs affects cognitive performance in AD mice, 3XTg-AD mice aged 12 months, an age of advanced cerebral pathology, were injected intrathymically (i.t.) with APP^−/−^ or APP^+/+^ mESC-TEPs. APP^−/−^ or APP^+/+^ mESC-derived EpCAM1^−^ non-TEPs (control cells) were used as controls. Two months later, the mice were evaluated for spatial learning and memory. It has been reported that the Barnes maze, a hippocampal-dependent spatial task (57, 58), is the most sensitive test for detecting cognitive deficits in 3XTg-AD mice (59). We found that both APP^−/−^ and APP^+/+^ mESC-TEP-treated mice had significantly greater Barnes maze learning curves than control cell-treated mice (Figure 2A). Furthermore, APP^−/−^ mESC-TEP-treated mice performed better than APP^+/+^ mESC-TEP-treated mice, almost reaching the performance level observed in wild-type (WT) non-AD mice (Figure 2A). Since there were no significant differences between APP^−/−^ and APP^+/+^ mESC-EpCAM1^−^ control cell-transplanted mice in all of the results in this paper (data not shown), we pooled the data from the two groups and named this group as control cell (Ctrl)-transplanted mice.

Both APP^−/−^ and APP^+/+^ mESC-TEP-treated mice also had decreased latency to find the target zone during the probe trial conducted 24 h after the final training session (Figure 2B), indicating an improved memory performance. In addition, the number of errors committed in the APP^−/−^ or APP^+/+^ mESC-TEP-treated mice was also significantly reduced, as compared to control cell-treated mice (Figure 2C).

The NOR test is to study learning and memory in rodents based on their spontaneous tendency to have more interactions with a novel than with a familiar object (60). NOR is a more cortically-dependent novel object recognition preference task (57, 58). In agreement with the results in the Barnes maze task, APP^−/−^ or APP^+/+^ mESC-TEP-treated mice performed significantly better than control cell-treated mice (Figure 2D). In all of these studies (Figures 2A–D), APP^−/−^ mESC-TEP-treated mice performed significantly better than APP^+/+^ mESC-TEP-treated mice. Together, our data suggest that transplantation of APP^−/−^ or APP^+/+^ mESC-TEPs results in improved spatial learning and memory in 3xTg-AD mice and that transplantation of APP^−/−^ mESC-TEPs demonstrates greater effectiveness than APP^+/+^ mESC-TEPs.

3. Both APP^−/−^ and APP^+/+^ mESC-TEP-transplanted AD mice have reduced AD pathology with greater reduction in APP^−/−^ mESC-TEP-transplanted mice.

**Figure 3:**
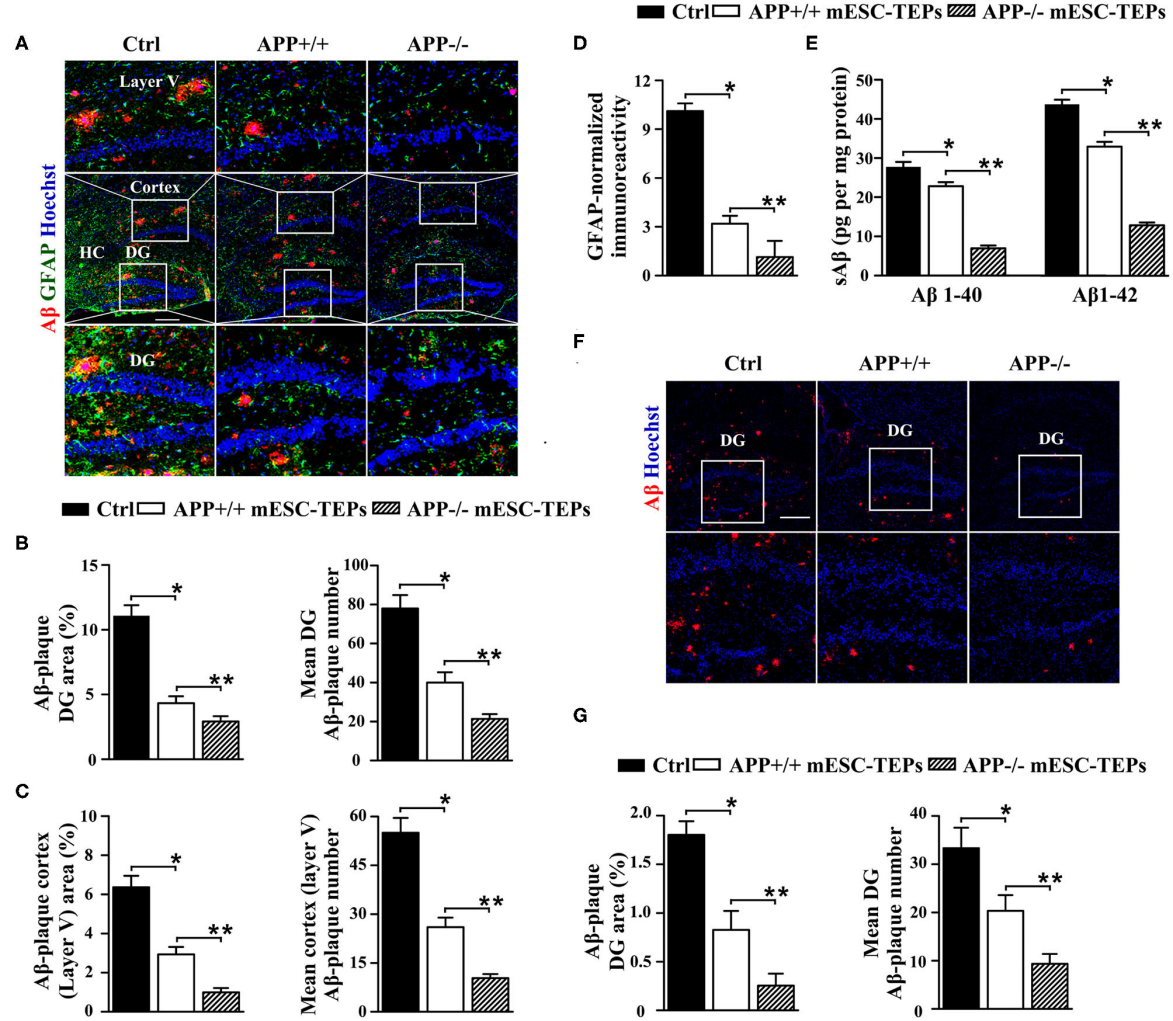
Transplantation of APP^−/−^ or APP^+/+^ mESC-TEPs attenuates AD pathology and APP^−/−^ mESC-TEPs are more effective than APP^+/+^ mESC-TEPs. **(A–C)** 3XTg-AD mice (12-month-old) were injected i.t. with APP^−/−^ mESC-TEPs, APP^+/+^ mESC-TEPs, or control cells as in Figure 2. Two and a half months later, the mice were evaluated for **(A–D)** brain pathology, and **(E)** soluble Aβ levels. **(A–D)** The brain sections were immunostained for Aβ (in red), GFAP (in green) and Hoechst nuclear staining. Mean Aβ area and plaque numbers in the hippocampal DG and the cortex fifth layer, and GFAP immunoreactivity in the hippocampus were measured. **(A)** Representative immunofluorescent images, and **(B–D)** quantitative analysis of Aβ and GFAP. **(E)** Levels of soluble Aβ1–40 and Aβ1–42 in the cerebral brain parenchyma of the mice were quantified by ELISA. **(F,G)** 14-month-old APP/PS1 mice were injected i.t. with APP^−/−^ mESC-TEPs, APP^+/+^ mESC-TEPs, or control cells. Two months later, the mice were evaluated for brain pathology. **(F)** Representative immunofluorescent images, and (G) quantitative analysis of Aβ. The data are expressed as mean ± SD from one of three independent experiments with similar results (four to eight mice per group per experiment). *p < 0.05 vs. control cell group, **p < 0.05 vs. APP^+/+^ mESC-TEP group.

We then determined whether transplantation of APP^−/−^ or APP^+/+^ mESC-TEPs leads to improved AD pathology. After the Barnes maze and NOR tests (Figure 2), the brains were harvested and immunohistochemical analysis performed. We found that both APP^−/−^ and APP^+/+^ mESC-TEP-transplanted 3XTg-AD mice had a reduced cerebral Aβ plaque load in the hippocampus, specifically in the dentate gyrus (DG) and in the cerebral cortex (layer V) (Figures 3A–C), areas showing robust Aβ-plaque pathology in AD mice. Comparatively, APP^−/−^ mESC-TEP-transplanted mice had the greater reduction in Aβ-plaque load (Figures 3A–C). Astrogliosis, as assessed by glial fibrillary acid protein (GFAP) immunoreactivity, was also reduced in APP^−/−^ and APP^+/+^ mESC-TEP-treated mice, as compared to control cell-treated mice (Figures 3A,D). Again, APP^−/−^ mESC-TEP-transplanted mice had, by comparison, a greater reduction in GFAP immunoreactivity (Figures 3A,D).

Since impaired synaptic plasticity and memory deficits in AD are associated with elevated cerebral soluble Aβ1-40/Aβ1-42 (sAβ) levels (61), we then measured sAβ levels in the AD mice by ELISA. Consistent with the immunohistochemical results, both APP^−/−^ and APP^+/+^ mESC-TEP-treated mice had reduced cerebral sAβ, as compared to control cell-treated mice (Figure 3E). Transplantation of APP^−/−^ mESC-TEPs demonstrated the greater reduction (Figure 3E).

We likewise examined the effect of mESC-TEPs in another AD model, APP/PS1 mice, which develop Aβ-plaque pathology at a more advanced age than do 3XTg-AD mice. Transplantation of APP^−/−^ or APP^+/+^ mESC-TEPs reduced hippocampal Aβ plaque load, as compared to control cell-treated mice, and APP^−/−^ mESC-TEP-treated mice had more Aβ plaque load reduction than APP^+/+^ mESC-TEP-treated mice (Figures 3F,G). Taken together, our results suggest that transplantation of APP^−/−^ or APP^+/+^ mESC-TEPs into AD mice results in clearance of Ab plaques and reversal of cognitive decline, and APP^−/−^ mESC-TEP-treated mice perform better than APP^+/+^ mESC-TEP-treated mice.

4. Both APP^−/−^ and APP^+/+^ mESC-TEP-transplanted AD mice have increased T cell numbers, and APP^−/−^ mESC-TEP-transplanted mice have enhanced T cell proliferation in response to Aβ stimulation.

**Figure 4:**
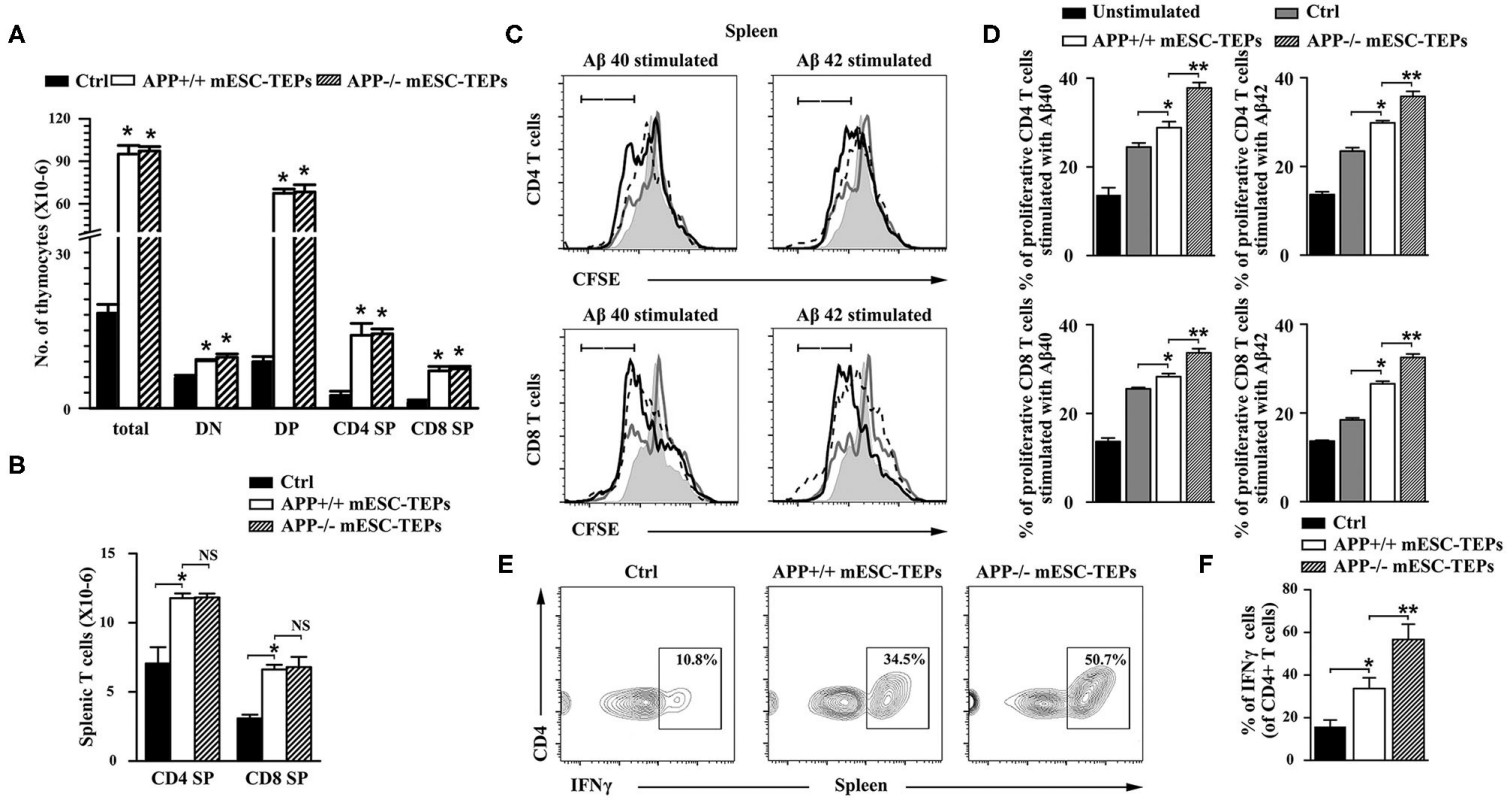
Transplantation of APP^+/+^ or APP^−/−^ mESC-TEPs leads to increased T cell number, and enhanced T cell proliferative response to Aβ proteins. 3XTg-AD mice (12-month-old) were injected i.t. with APP^−/−^ mESC-TEPs, APP^+/+^ mESC-TEPs, or control cells as in Figure 2. Two and a half months later, the thymus and spleen were harvested. The number of **(A)** total thymocytes and their subsets, and **(B)** CD4 and CD8 T cells in the spleen were analyzed by flow cytometry. **(C,D)** The splenocytes (normalized to 1 × 10^5^ T cells/well) from the AD mice were labeled with CFSE and cultured with Aβ40 or Aβ42 in the presence of anti-CD3 antibody for 3 days. The cells were stained with mouse anti-CD4 and CD8 antibodies and analyzed for CFSE levels by CD4^+^ and CD8^+^ T cells, respectively. Black line: APP^−/−^ mESC-TEP-transplanted mice; black dash line: APP^+/+^ mESC-TEP-transplanted mice; gray line: control cell-transplanted mice; gray shadow: unstimulated T cells. **(E,F)** The splenocytes were analyzed for the percentage of IFNγ-producing CD4 T cells. **(C,E)** Representative flow cytometric profiles, and **(D,F)** statistical analyses are shown. The data are expressed as mean ± SD from one of three independent experiments with similar results (4–8 mice per group in each experiment). *p < 0.05 vs. control cell group, **p < 0.05 vs. APP^+/+^ mESC-TEP group.

We have previously demonstrated that transplantation of mESC-TEPs results in enhanced thymopoiesis and increased T cell numbers in the spleen (43–45). Consistent with the previous reports, APP^+/+^ or APP^−/−^ mESC-TEP-transplanted mice had increased numbers of thymocytes in the thymus and T cells in the spleen compared to control cell-treated mice. The number of T cells in the thymus and the spleen between APP^+/+^ and APP^−/−^ mESC-TEP-transplanted mice were not significantly different (Figures 4A,B).

Thymocytes can be divided into four major subsets: CD4 and CD8 double negative (DN), double positive (DP), CD4 single positive (SP), and CD8 SP thymocytes. DN thymoctyes can be further divided into DN1 to DN4 subsets based on the expression of CD44 and CD25. APP^+/+^ or APP^−/−^ mESC-TEP-transplanted mice had decreased percentages of DN subsets (Supplemental Figure 3), suggesting improved thymocyte development. Because APP^+/+^ or APP^−/−^ mESC-TEP-transplanted mice had increased numbers of total thymocytes, and numbers of each thymocyte subsets in these mice were higher than those in control cell-treated mice (Figure 4A and Supplemental Figure 3). We also analyzed the percentage and number of regulatory T cells (Tregs) in the thymus and the spleen. Although the percentages of Tregs were not significant different among the groups, the number of Tregs in APP^+/+^ or APP^−/−^ mESC-TEP-transplanted AD mice was higher than WT or control cell-transplanted AD mice (Supplemental Figures 3, 4). Similarly, the percentages of CD11c^+^ dendritic cells (DCs) and CD19^+^ B cells in the spleen were not significant different among the groups (Supplemental Figure 4).

We then determined the proliferation of the splenic T cells in response to Aβ40 and Aβ42 protein stimulation in the presence of anti-CD3 antibody *in vitro*. The proliferation of CD4 and CD8 T cells from APP^+/+^ mESC-TEP-transplanted mice was slightly higher than that from control cell-transplanted mice (Figures 4C,D), which may be due to enhanced T cell function after transplantation of mESC-TEPs. Furthermore, the proliferation of both CD4 and CD8 T cells from APP^−/−^ mESC-TEP-transplanted mice was significantly higher than that from APP^+/+^ mESC-TEP-transplanted mice (Figures 4C,D). The latter results suggest that Aβ-specific autoreactive T cells might not be deleted in the thymus of APP^−/−^ mESC-TEP-transplanted mice, leading to presence of Aβ-specific autoreactive T cells in the periphery, resulting in greater proliferation response. Of note, although the proliferation of both CD4 and CD8 T cells in response to anti-CD3 antibody alone (without Aβ40 or Aβ42 peptide) from APP^+/+^ or APP^−/−^ mESC-TEP-transplanted mice was greater than that from control cell-transplanted mice, there was no significant difference between APP^+/+^ and APP^−/−^ mESC-TEP-transplanted groups (data not shown).

It has been reported that PD-1 blockage reduced AD pathology involves an IFNγ-dependent immunological response (4). We then analyzed IFNγ-producing T cells in the spleen and found that the percentage of IFNγ-producing CD4 T cells in APP^+/+^ or APP^−/−^ mESC-TEP-transplanted AD mice was significantly higher than that in control cell-treated mice (Figures 4E,F). This is consistent with our previous reports that transplantation of mESC-TEPs leads to the generation of functional T cells including enhanced production of IFNγ (44, 45). Compared with APP^+/+^ mESC-TEP-transplanted mice, the percentage of IFNγ-producing CD4 T cells in APP^−/−^ mESC-TEP-transplanted mice showed a larger increase, which is probably due to enhanced anti-Aβ autoimmunity in APP^−/−^ mESC-TEP-transplanted AD mice. Furthermore, more IFNγ was detected in the supernatant of cultured splenocyts from APP^−/−^ mESC-TEP-transplanted AD mice in response to Aβ40 or Aβ42 protein stimulation (Supplemental Figure 5).

5. APP^−/−^ mESC-TEP-transplanted mice have an increased number of anti-Aβ Ab-producing B cells in the spleen and increased level of anti-Aβ Ab in the serum.

**Figure 5:**
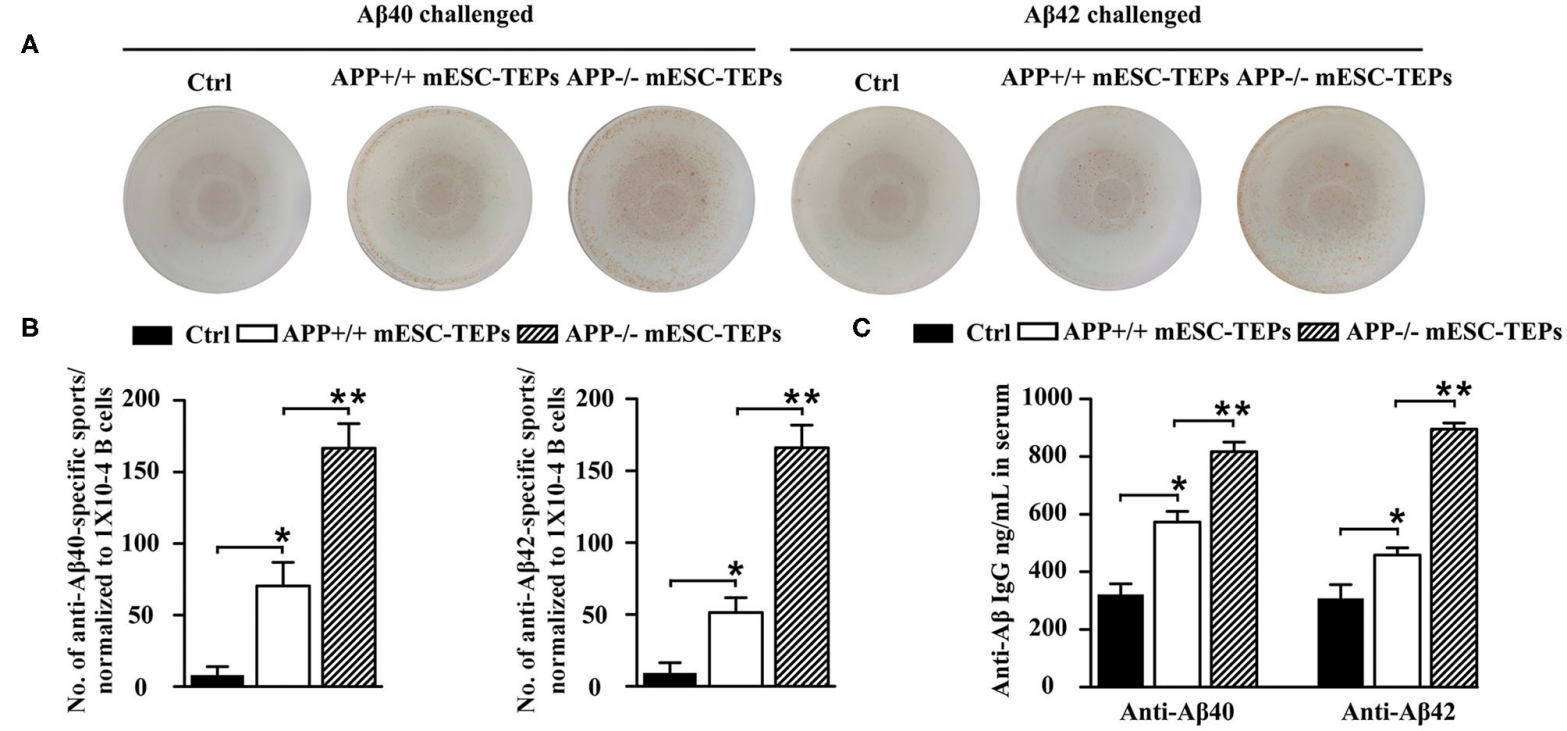
Transplantation of APP^−/−^ mESC-TEPs results in an increased number of anti-Aβ Ab-producing B cells in the spleen and increased levels of anti-Aβ Abs in the serum. 3XTg-AD mice were injected i.t. with APP^−/−^ mESC-TEPs, APP^+/+^ mESC-TEPs, or control cells as in Figure 2. Two and half months later, the spleen and serum were harvested. **(A,B)** The splenocytes (normalized to 1 × 10^4^ CD19^+^ B cells/well) were placed on culture plates coated with Aβ40 (4 μg/ml) or Aβ42 (4 μg/ml). Anti-Aβ Ab-producing B cells were measured by an ELISpot assay. **(C)** The levels of anti-Aβ40 and anti-Aβ42 antibodies in the serum were measured by ELISA. The data are expressed as mean ± SD from one of three independent experiments with similar results (4–8 mice per group in each experiment). *p < 0.05 vs. control cell group, **p < 0.05 vs. APP^+/+^ mESC-TEP group.

It is well-known that T cells can help B cell functions. We then determined whether the enhanced T cell proliferation to Aβ stimulation in mESC-TEP-transplanted AD mice led to increased production of anti-Aβ Ab-producing B cells. We used Aβ40 and Aβ42 as antigens for an ELISpot assay to measure anti-Aβ Ab-producing B cells in the spleen. The number of anti-Aβ Ab-producing B cells in APP^+/+^ mESC-TEP-transplanted mice was higher than that in control cell-transplanted mice, while the number of anti-Aβ Ab-producing B cells in APP^−/−^ mESC-TEP- transplanted mice was higher than that in APP^+/+^ mESC-TEP-transplanted mice (Figures 5A,B).

We also analyzed the levels of anti-Aβ Abs in the serum. Consistent with the ELISpot results, the levels of both anti-Aβ40 and anti-Aβ42 antibodies in the serum of APP^+/+^ mESC-TEP-treated mice were higher than those in control cell-treated mice, and the levels of these antibodies in APP^−/−^ mESC-TEP-treated mice were significantly higher than those in APP^+/+^ mESC-TEP-treated mice (Figure 5C). The results suggest that increased T cell number in APP^+/+^ or APP^−/−^ mESC-TEP-treated mice, especially Aβ-specific T cells in APP^−/−^ mESC-TEP-treated mice, help to generate anti-Aβ Ab-producing B cells that secret anti-Aβ Abs into the serum.

6. Transplantation of both APP^−/−^ and APP^+/+^ mESC-TEPs enhances the brain's choroid plexus (CP) activity.

**Figure 6:**
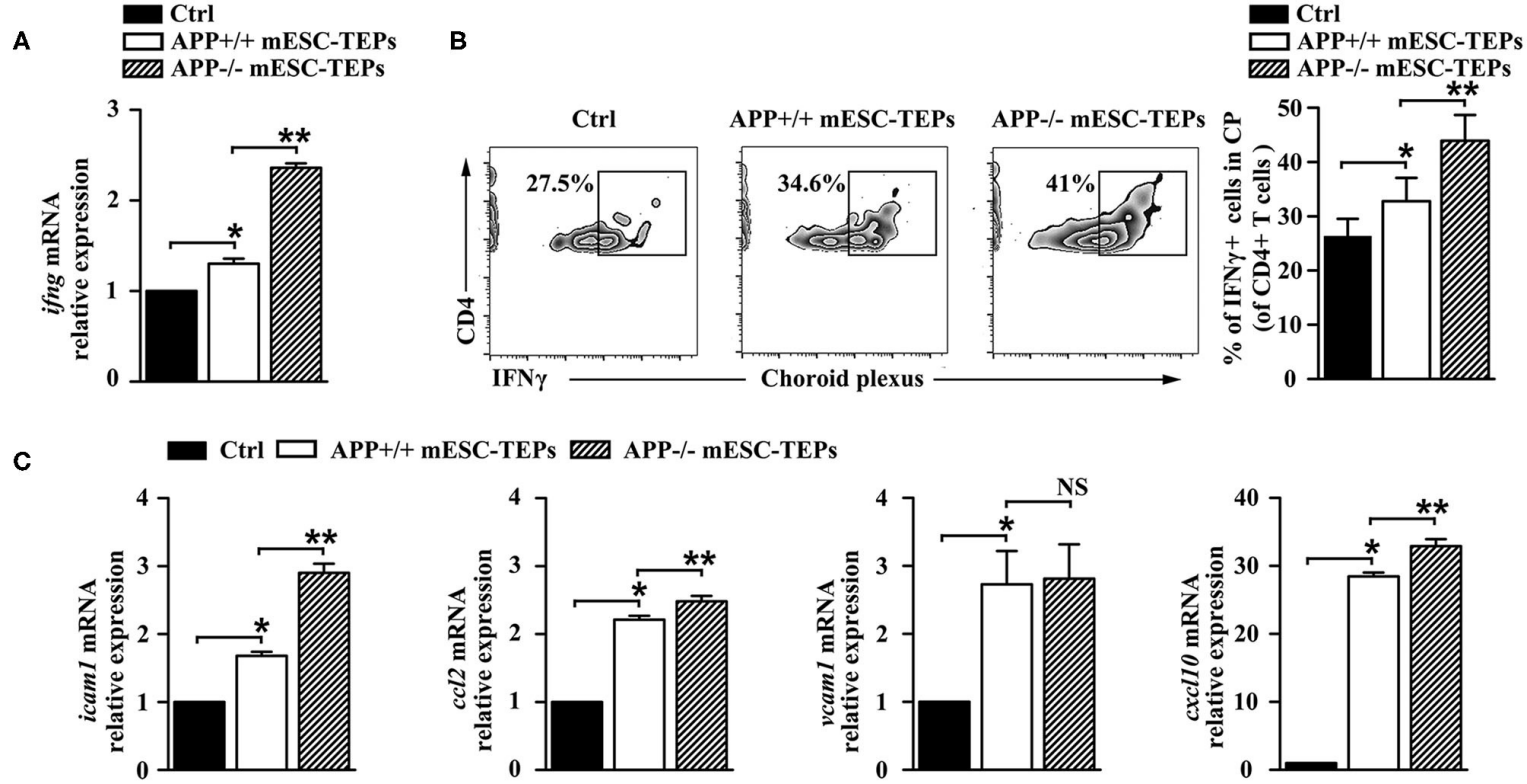
Transplantation of APP^−/−^ or APP^+/+^ mESC-TEPs increases IFNγ availability and leukocyte trafficking molecule expression in the CP. 3XTg-AD mice were injected i.t. with APP^−/−^ mESC-TEPs, APP^+/+^ mESC-TEPs, or control cells as in Figure 2. Two and half months later, the CP was harvested. **(A)** mRNA levels of IFNγ were analyzed by qRT-PCR. **(B)** Flow cytometric analysis for the percentage of IFNγ-producing cells in CD4^+^ cells, (left) representative flow cytometric profiles, and (right) statistical analyses. **(C)** mRNA levels of icam1, ccl2, vcam1, cxcl10 were analyzed by qRT-PCR. The expression levels of the genes in control cell-treated mice are defined as 1. The data are expressed as mean ± SD from one of three independent experiments with similar results (4–8 mice per group in each experiment). *p < 0.05 vs. control cell group, **p < 0.05 vs. APP^+/+^ mESC-TEP group.

The CP, the epithelial layer that forms the blood–CSF barrier, is a selective gateway for leukocyte entry to the CNS (13). AD mice have a defect in the CP gateway, as indicated by significantly lower levels of leukocyte homing and trafficking molecule expression in the CP (13). In contrast, IFNγ signaling enhances the expression of leukocyte trafficking molecules (15). Since we have demonstrated that IFNγ-producing T cells were increased in the spleen of APP^−/−^ or APP^+/+^ mESC-TEP-treated AD mice (Figure 4E), we analyzed IFNγ availability at the CP in these mice. qRT-PCR analysis revealed a higher IFNγ mRNA expression level in the CP of APP^−/−^ or APP^+/+^ mESC-TEP-transplanted AD mice, compared with control cell-treated mice (Figure 6A). Flow cytometric examination confirmed a significantly higher percentage of IFNγ-producing CD4^+^ immune cells in this compartment in APP^−/−^ or APP^+/+^ mESC-TEP-transplanted AD mice (Figure 6B). Again, the IFNγ mRNA expression levels and the percentage of IFNγ-producing CD4^+^ immune cells in the CP APP^−/−^ mESC-TEP-transplanted AD mice were higher than those in APP^+/+^ mESC-TEP-transplanted AD mice (Figures 6A,B), consistent with the results for the percentage of IFNγ-producing CD4^+^ splenic T cells among the mice (Figure 4E).

Since increased IFNγ availability can enhance CP activity in this compartment (4, 13), we analyzed the expression of leukocyte homing and trafficking molecules, including intercellular adhesion molecule 1 (icam1), chemokine C-C motif ligand 2 (ccl2), vascular cell adhesion molecule 1 (vcam1), and C-X-C motif chemokine 10 (cxcl10) in the CP. As shown in Figure 6C, the mRNA expression levels of these leukocyte trafficking molecules in the CP of APP^+/+^ mESC-TEP-treated AD mice were higher than those in control cell-treated mice. The mRNA expression levels of these molecules (except vcam1) in the CP of APP^−/−^ mESC-TEP-treated AD mice were higher than those in APP^+/+^ mESC-TEP-treated mice (Figure 6C). These results suggest that administration of APP^−/−^ or APP^+/+^ mESC-TEPs results in an increased CP activity, which is likely due to the increased IFNγ availability in this compartment.

7. APP^−/−^ mESC-TEP-transplanted mice have an increased number of Aβ phagocytosing macrophages in the brain and the spleen.

**Figure 7:**
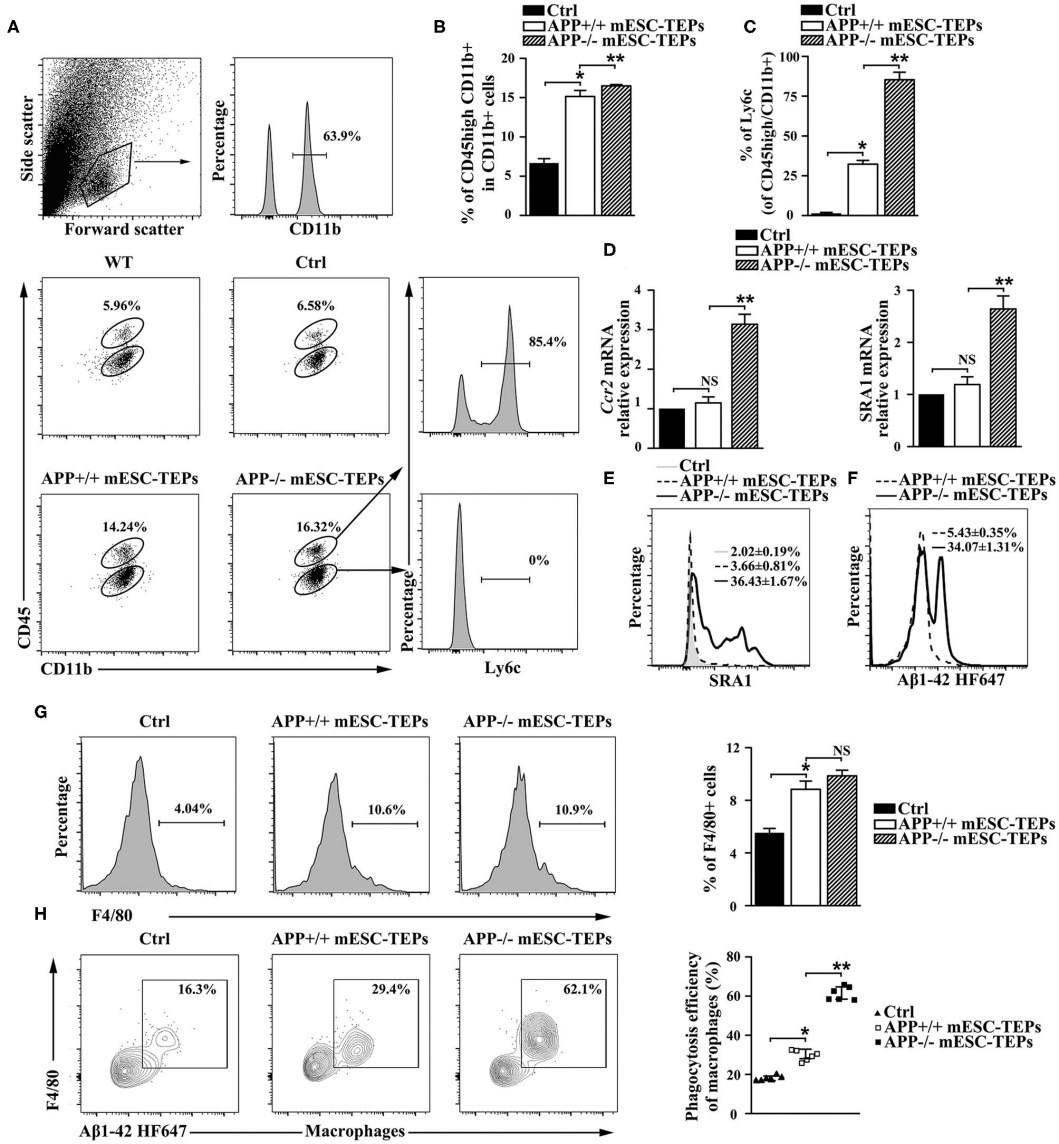
Transplantation of APP^−/−^ mESC-TEPs results in an increased number of Aβ phagocytosing macrophages in the brain and the spleen. 3XTg-AD mice were injected i.t. with APP^−/−^ mESC-TEPs, APP^+/+^ mESC-TEPs or control cells as in Figure 2. Two and half months later, **(A–F)** the brain and **(G,H)** the spleen were harvested. **(A–C)** The brain was analyzed for the percentage of CD45^hi^/CD11b^+^ cells and the expression of Ly6C by CD45^hi^ or CD45^lo^ cells. **(A)** Flow cytometry gating strategy is shown. **(B,C)** Statistical analysis of the percentages of **(B)** CD45^hi^/CD11b ^+^ cells in CD11b ^+^ cells and **(C)** Ly6C^+^ in CD45^hi^/CD11b ^+^ cells. **(D,E)** The brain was analyzed for **(D)** the expression of ccr2 and SRA1 mRNA by qRT-PCR (the expression level of the genes in control cell-treated mice is defined as 1), and **(E)** the expression of SRA1 protein by CD45^hi^CD11b ^+^ cells using flow cytometry. **(F)** CD45^hi^/CD11b ^+^ cells were isolated from brains and analyzed for phagocytosis using HF647 Aβ42. **(G,H)** The splenocytes were analyzed for **(G)** the percentage of F4/80^+^ macrophages and **(H)** the ability of F4/80^+^ macrophages to phagocytose Aβ42. The data are expressed as mean ± SD from one of three independent experiments with similar results (4–8 mice per group in each experiment). *p < 0.05 vs. control cell group, **p < 0.05 vs. APP^+/+^ mESC-TEP group.

Increased CP activity can result in recruitment of monocyte-derived macrophages to the brain to attenuate AD pathology (4, 13). Since transplantation of APP^−/−^ or APP^+/+^ mESC-TEPs led to an increased CP activity, we analyzed whether there was an increased number of monocyte-derived macrophages in the brain. It has been shown that CD45^hi^/CD11b^+^ cells represent a myeloid population enriched with CNS-infiltrating monocyte-derived macrophages in the brain (4, 13). We found both APP^−/−^ and APP^+/+^ mESC-TEP-transplanted AD mice had an elevated proportion of CD45^hi^/CD11b^+^ cells in the brain, as compared to control cell-treated mice (Figures 7A,B). The proportion of CD45^hi^/CD11b^+^ cells in the brain of APP^−/−^ mESC-TEP-transplanted AD mice was higher than that in APP^+/+^ mESC-TEP-transplanted mice. Furthermore, the CD45^hi^/CD11b^+^ cells in APP^−/−^ mESC-TEP-transplanted mice had a higher percentage of lymphocyte antigen 6c (Ly6C) positive cells than those in APP^+/+^ mESC-TEP-transplanted mice (Figure 7C). The CD45^hi^/CD11b^+^ cells in APP^−/−^ mESC-TEP-transplanted mice also expressed higher levels of chemokine receptor ccr2 and scavenger receptor A (SRA1) (Figures 7D,E). It has been reported that Ly6C and ccr2 are related to myeloid cell neuroprotection in AD (62), whereas SRA1 is an Aβ-binding scavenger receptor associated with Aβ-plaque clearance (63). We also analyzed the phagocytosis ability of CD45^hi^/CD11b^+^ cells and found that the cells in APP^−/−^ mESC-TEP-transplanted mice had a higher ability to phagocytose Aβ42 than those in APP^+/+^ mESC-TEP-transplanted mice (Figure 7F).

We then analyzed macrophages in the spleen and found the percentages of F4/80^+^ macrophages in APP^−/−^ or APP^+/+^ mESC-TEP-transplanted AD mice were higher than that those in control cell-treated mice (Figure 7G). Although the percentage of F4/80^+^ macrophages in APP^−/−^ mESC-TEP-transplanted mice was slightly higher than that in APP^+/+^ mESC-TEP-transplanted mice, the difference did not reach statistical significance (Figure 7G). Furthermore, F4/80^+^ macrophages in APP^+/+^ mESC-TEP-transplanted mice were more able to phagocytose Aβ42 than those in control cell-transplanted mice, and the macrophages in APP^−/−^ mESC-TEP-transplanted mice had a greater ability than those in APP^+/+^ mESC-TEP-transplanted mice (Figure 7H), in agreement with the data for the macrophage function in the brain (Figure 7F).

## Discussion

AD is hallmarked by the accumulation of Aβ plaques in the brain, which can adversely affect synaptic function and eventually cause neuron loss (2–4, 7, 8). The brain has been traditionally considered a site of immune privilege and exempt from systemic immune surveillance (16, 24). It is now accepted that neuro-immunological cross-talk, in which circulating immune cells enter the CNS, play an important role in brain tissue maintenance and repair, especially in pathological conditions (18, 24–26, 64–66). Like the situation in cancer immunology, onset of AD may reflect systemic immune suppression and the loss of immune surveillance (13, 67), impairing the ability to mount an immune response to fight brain pathology (13, 14, 68). For example, it has been shown that AD severity is greater in immunocompromised mice (19). In contrast, replacement of the missing adaptive immune populations, such as T cells and B cells, can dramatically reduce AD pathology (19). Boosting recruitment of monocyte-derived macrophages to sites of brain pathology also facilitates Aβ plaque clearance and relieves AD pathology (4, 13, 69–72). Therefore, systemic immunity in AD should be driven, rather than suppressed, to initiate an immune-dependent cascade to dissipate the Aβ clearance and repair the brain (4, 13, 14).

It is well-known that the thymus, the primary organ for T cell generation, undergoes a profound atrophy with age, a process termed thymic involution, resulting in decreased numbers of T cells in older adults. The reduced T cell number in older adults is likely to contribute AD development and progression. Indeed, in this study, we have shown that transplantation of either APP^−/−^ or APP^+/+^ mESC-TEPs enhances thymopoiesis that results in increased number of T cells, especially IFNγ-producing T cells in the spleen and the CP, leading to enhanced CP activity and increased number of macrophages in the brain. In addition, these mice also have an increased number of macrophages in the spleen. It has been shown that increased IFNγ availability in the CP can enhance the CP activity (4, 13). Both APP^+/+^ and APP^−/−^ mESC-TEP-transplanted mice have enhanced expression of leukocyte homing and trafficking molecules icam1, vcam1, cxcl10, and ccl2 in the CP, which may be due to the increased IFNγ availability in this compartment. It is likely that the enhanced CP activity leads to increased migration of macrophages into the brain, resulting in an increased number of macrophages in this organ. It is also possible that increased T cell numbers in the spleen aid the macrophages, increasing their number in the spleen, likewise contributing to the increase in the brain. Although the improved thymopoiesis and an increased number of immune cells in the periphery (especially macrophages in the brain) attenuate AD pathology, they are insufficient for reduction of cerebral Aβ plaque load and for improving cognitive performance as indicated by the data that transplantation of APP^+/+^ mESC-TEPs is less efficient than that of APP^−/−^ mESC-TEPs.

Compared to APP^+/+^ mESC-TEP-transplanted mice, APP^−/−^ mESC-TEP-transplanted mice have increased Aβ-induced T cell proliferation, increased anti-Aβ Ab-producing B cells in the spleen and anti-Aβ Abs in the serum, as well as increased Aβ phagocytosing macrophages in the brain. Since TECs expressing self-antigens play a critical role in deleting autoreactive T cells specific to the antigens, transplantation of APP^−/−^ mESC-TEPs could result in the failure to delete Aβ-specific autoreactive T cells in the thymus, leading to the presence of the autoreactive T cells in the periphery. This is supported by the data that T cells from APP^−/−^ mESC-TEP-transplanted mice have increased proliferation in response to Aβ stimulation. The Aβ-specific autoreactive T cells may then help to produce anti-Aβ Ab-producing B cells that secret anti-Aβ Abs into the serum and to produce Aβ phagocytosing macrophages that migrate into the brain. Together, these Aβ-specific immune cells and Abs reduce the AD pathology. Our results support the notion that breaking Aβ-specific immune tolerance is a novel target for AD immunotherapy (14).

Studies have shown that adaptive–innate immunity cross talk is important in ameliorating AD progression, in which T cells are critical (19). CD4 T cells are essential in the activation of B cells to secrete antibodies to mediate humoral immune responses (73, 74). Antibody response to an antigen requires help from the antigen-specific T cells. B cell antigen receptor usually delivers an antigen to intracellular sites where it is degraded and returned to the B cell surface as the peptide bound to MHC II molecule. The peptide:MHC II complex is recognized by the antigen-specific helper T cells, inducing the B cells to develop into antibody-secreting cells. It is possible that Aβ-specific autoreactive T cells generated in APP^−/−^ mESC-TEP-transplanted AD mice recognize the Aβ peptide:MHC II on B cells, and stimulate the B cells to proliferate and differentiate into plasma cells secreting anti-Aβ antibodies. Consequently, the anti-Aβ antibodies neutralize the toxin of Aβ and/or facilitate uptake of Aβ by macrophages by coating to Aβ to enhance the recognition by Fc receptors on macrophages.

CD4 T cells are also important in activating macrophages (75). Once activated, the macrophages phagocytose the related antigens. It has been shown that recruitment of circulating monocyte-derived macrophages can modify AD pathology (16, 76–78) by removing misfolded protein including Aβ-plaques (69, 79, 80), balancing the local inflammatory milieu (71, 80), reducing gliosis (81), and protecting synaptic structures (71, 82, 83). Since activated macrophages can cause local tissue damage (84–87), it is important that the macrophage activity is strictly regulated by antigen-specific T cells. It will be of interest to determine whether Aβ-specific autoreactive T cells in APP^−/−^ ESC-TEP-transplanted AD mice only activate the macrophages that specifically phagocytose Aβ, avoiding unnecessary local tissue damage.

**Figure 8:**
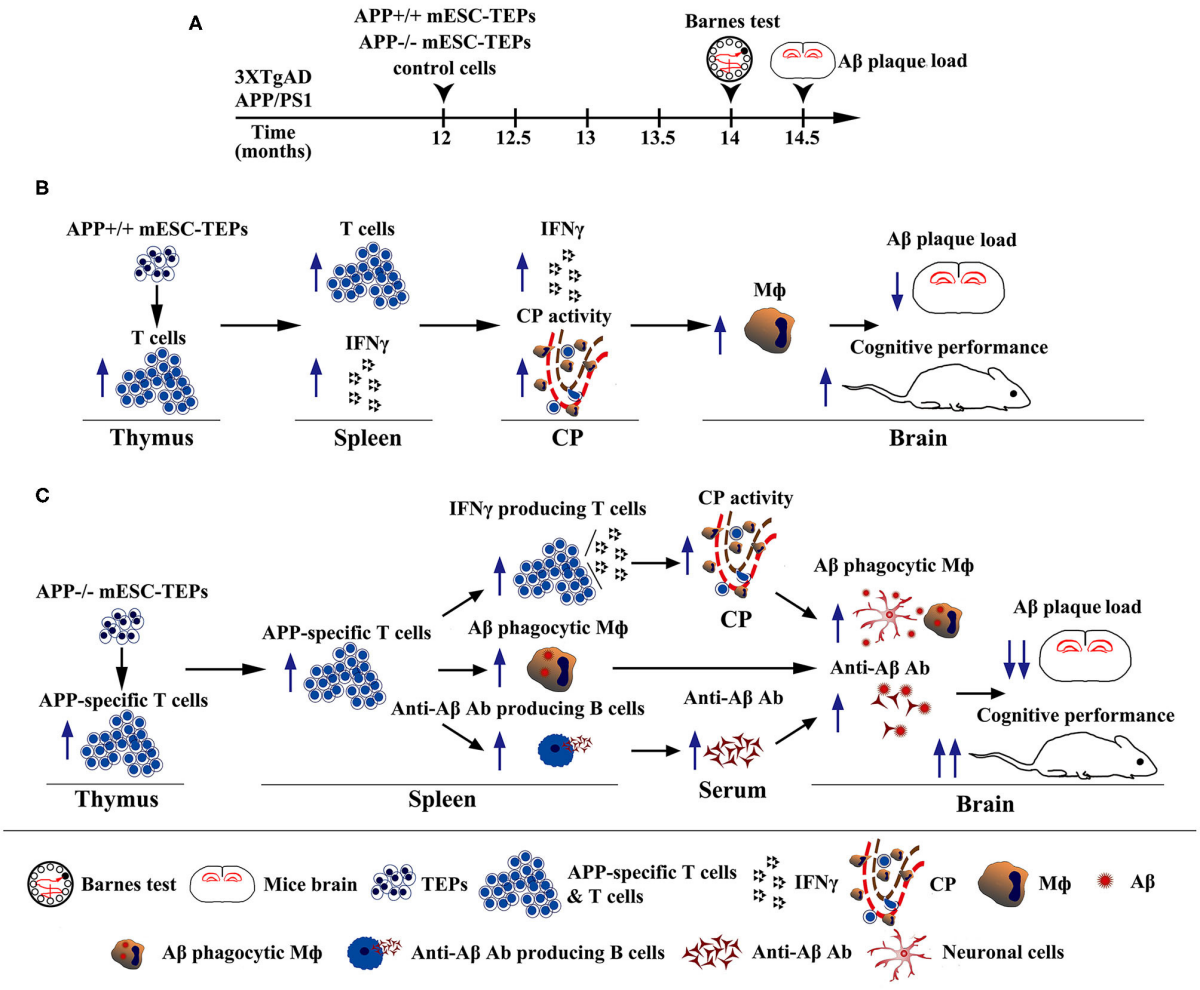
Diagrams showing the mechanisms by which transplantation of APP^+/+^ or APP^−/−^ mESC-TEPs into AD mice attenuates AD pathology. **(A)** Schematic of transplantation of mESC-derived cells and analyses of the AD mice. **(B,C)** Diagrams of the mechanisms of transplantation of **(B)** APP^+/+^ or **(C)** APP^−/−^ mESC-TEPs into AD mice reduces Aβ plaque load and increases cognitive performance.

In summary, we have demonstrated that transplantation of APP^+/+^ or APP^−/−^ mESC-TEPs into AD mice attenuates AD pathology, which is associated with enhanced systemic IFNγ-producing T cells and CP gateway activity with increased expression levels of leukocyte homing and trafficking molecules, as well as increased number of macrophages in the CNS. Furthermore, transplantation of APP^−/−^ mESC-TEPs has significantly greater effectiveness. This is related to the generation of T cells reactive with Aβ, which accompanied by increased number of anti-Aβ Ab-producing B cells in the spleen and enhanced level of anti-Aβ Ab in the serum, as well as an increased number of Aβ phagocytosing macrophages in the brain (Figure 8). Our results suggest that transplantation of APP^−/−^ human ESC-TEPs or iPSC-TEPs has the potential to be used in the prevention and treatment of AD patients.

## Data Availability Statement

The datasets generated for this study are available on request to the corresponding author.

## Ethics Statement

The animal study was reviewed and approved by Institutional Animal Care and Use Committee of the University of Connecticut.

## Author Contributions

JZ designed experiments, performed experiments, analyzed data, and wrote the manuscript. MS performed experiments and analyzed data. YL and HL performed experiments. ZH designed experiments, analyzed data, and supervised the study. LL designed experiments, analyzed data, supervised the study and wrote the manuscript. All authors contributed to the article and approved the submitted version.

## Conflict of Interest

The authors declare that the research was conducted in the absence of any commercial or financial relationships that could be construed as a potential conflict of interest.

## Abbreviations

AD: Alzheimer's disease
Aβ: amyloid-beta
CNS: central nervous system
TECs: thymic epithelial cells
ESCs: embryonic stem cells
TEPs: thymic epithelial progenitors
CP: choroid plexus
APP: e amyloid precursor protein
Ab: antibody
iPSCs: induced pluripotent stem cell
CRISPR: clustered Regularly Interspaced Short Palindromic Repeats
Cas9: CRISPR-associated protein
qRT-PCR: real-time qualitative RT-PCR
K5: keratin 5
K8: keratin 8
NOR: Novel object recognition
sAβ: soluble Aβ protein
i.t.: intrathymically
WT: wild-type
Ctrl: control cell
DG: dentate gyrus
GFAP: glial fibrillary acid protein
icam1: intercellular adhesion molecule 1
ccl2: chemokine C-C motif ligand 2
vcam1: vascular cell adhesion molecule 1
cxcl10: C-X-C motif chemokine 10
SRA1: scavenger receptor A.
