# Supplementary material for: Administration of Amyloid Precursor Protein Gene Deleted Mouse ESC-Derived Thymic Epithelial Progenitors Attenuates Alzheimer's Pathology
Source: Front Immunol. 2020 Aug 11;11:1781. doi: 10.3389/fimmu.2020.01781 (PMC7431620; doi:10.3389/fimmu.2020.01781)
Supplement: Supplementary file 1 [file Data_Sheet_1.PDF]

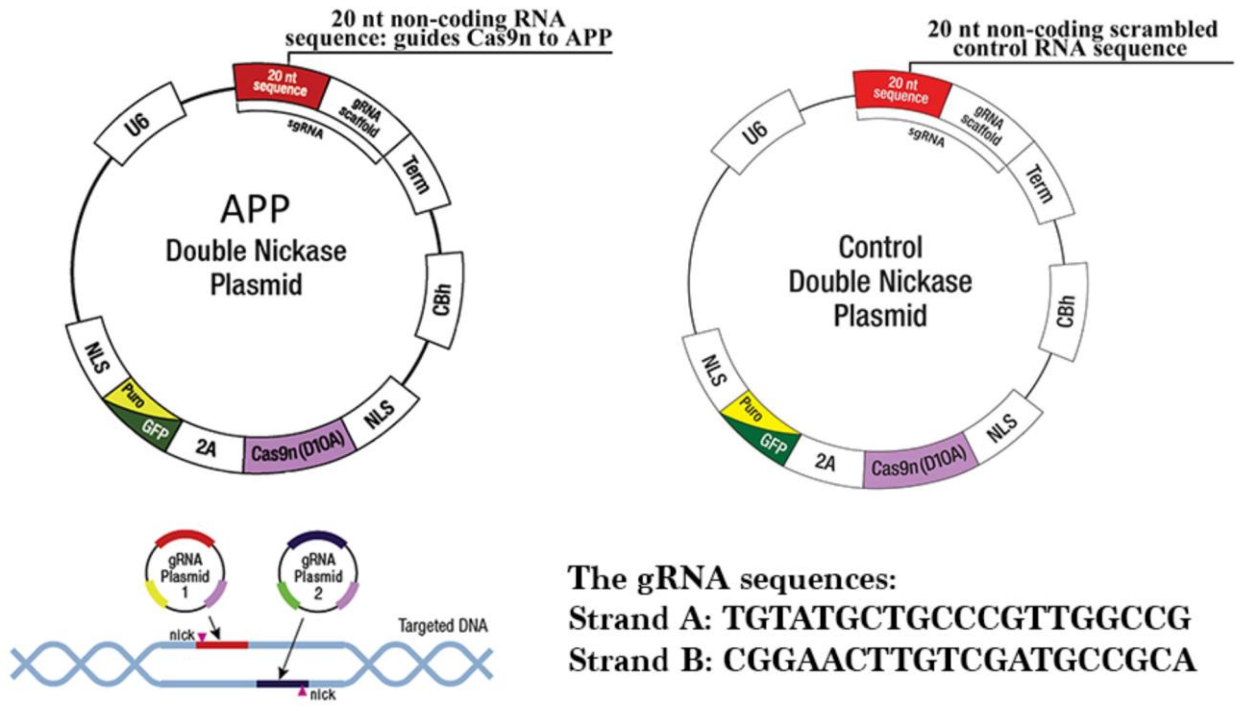

Supplemental Figure 1. The maps for APP-specific double nickase and control double nickase plasmids, and the APP gRNA sequences

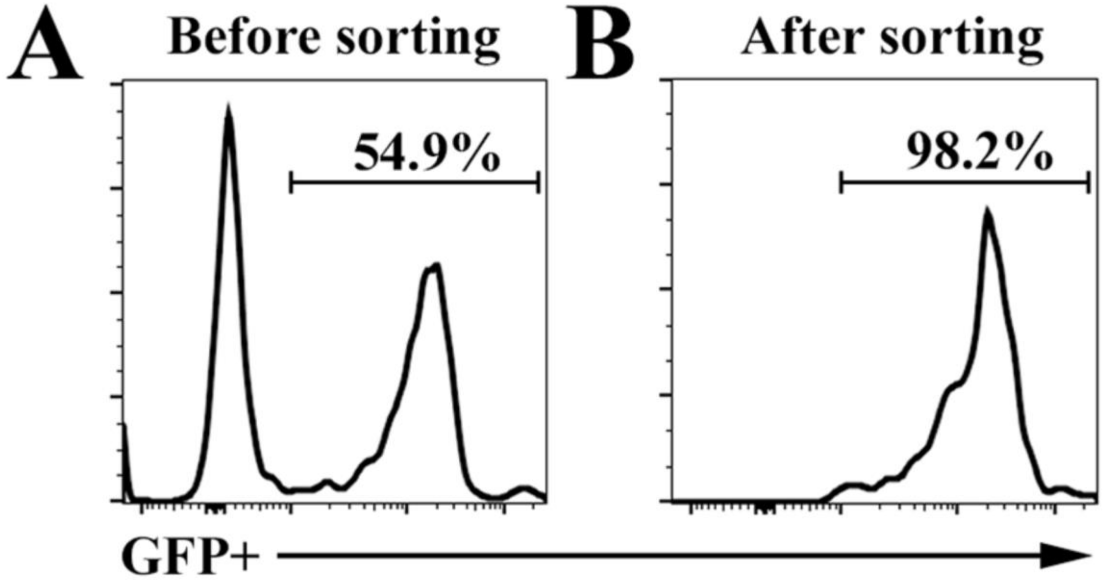

Supplemental Figure 2. Flow cytometry profiles of GFP^+ TECs in total TECs (A) before and (B) after cell sorting

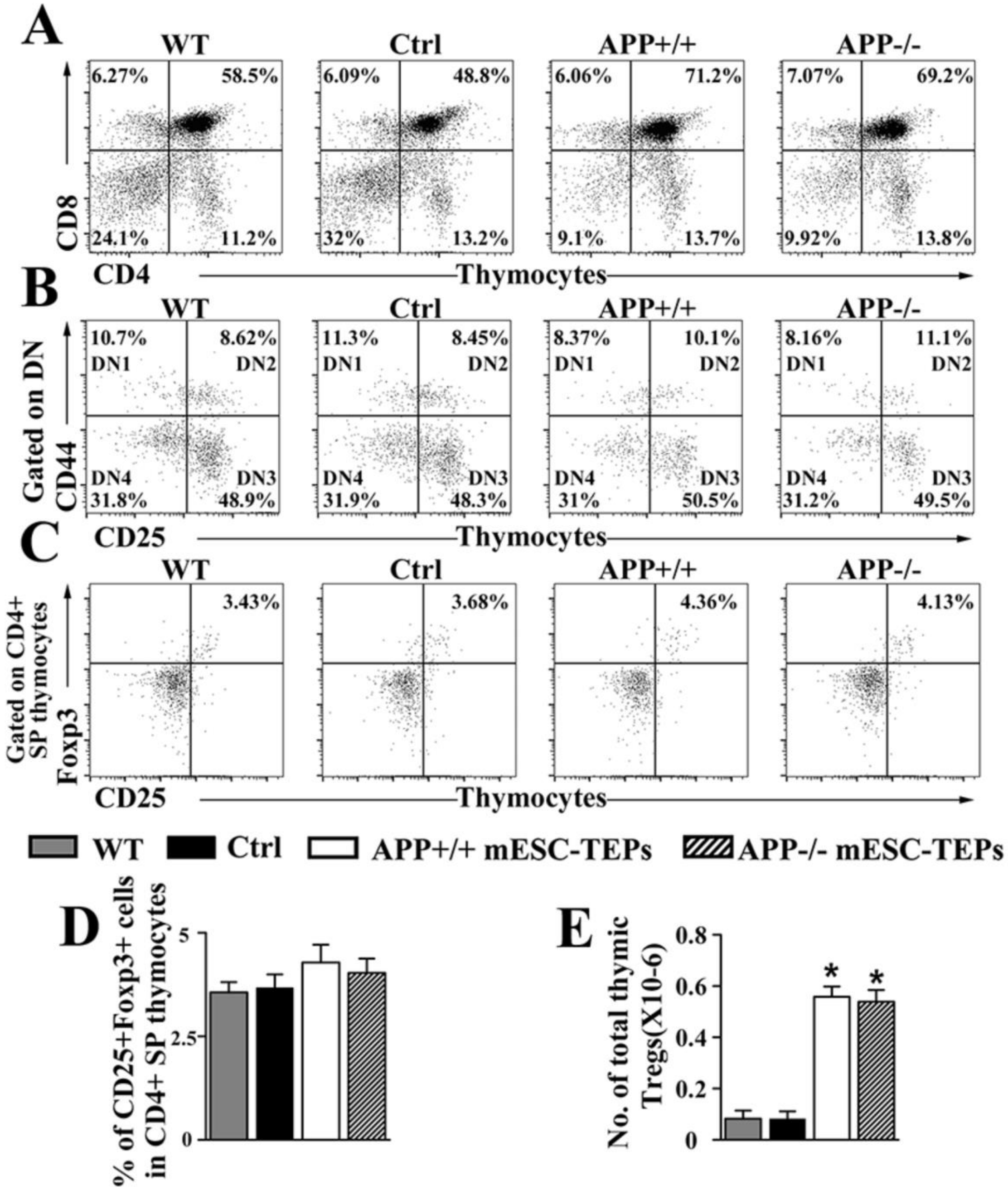

Supplemental Figure 3. The percentages of thymocyte subsets in WT-, control cell-, $APP^{+/+}$ or $APP^{-/-}$ mESC-TEP-transplanted AD mice. 3XTg-AD mice (12-month-old) were injected i.t. with $APP^{-/-}$ mESC-TEPs, $APP^{+/+}$ mESC-TEPs, or control cells as in Figure 2. Two and a half months later, the thymi were harvested and the percentages of thymocyte subsets were analyzed by flow cytometry. The percentages of (A) CD4 and CD8 DN, DP and SP subsets, and (B) DN1-DN4 subsets, as well as (C-E) the percentage and number of regulatory T cells. (A-C) Representative flow cytometric profiles, and (D, E) statistical analyses are shown. The data are expressed as mean ± SD from one of three independent experiments with similar results (4-8 mice per group in each experiment). *$p < 0.05$ versus control cell group.

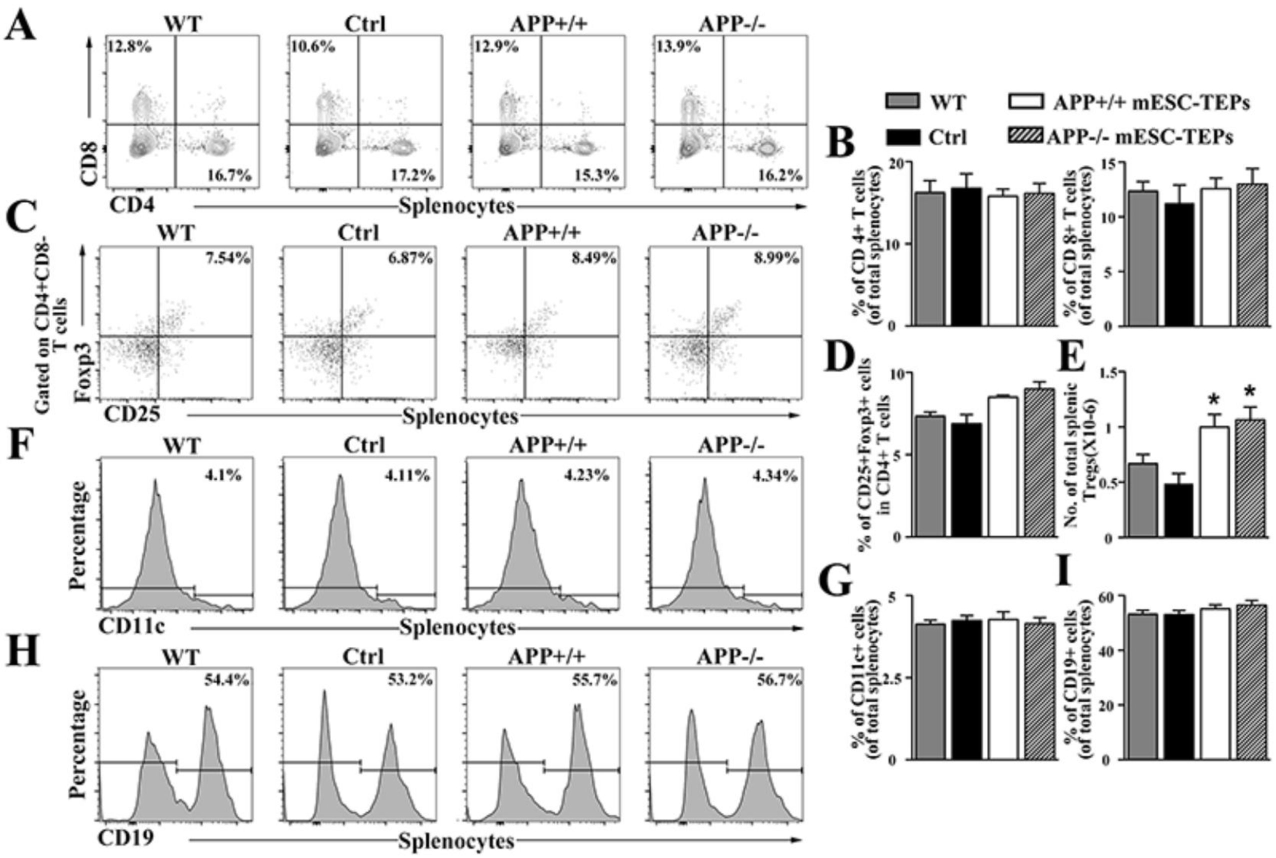

Supplemental Figure 4. The percentages of immune cells in WT-, control cell-, APP$^{+/+}$ or APP$^{-/-}$ mESC-TEP-transplanted AD mice. 3XTg-AD mice (12-month-old) were injected i.t. with APP$^{-/-}$ mESC-TEPs, APP$^{+/+}$ mESC-TEPs, or control cells as in Figure 2. Two and a half months later, the spleens were harvested and the percentages of immune cells were analyzed by flow cytometry. The percentage of (A, B) CD4^{+} and CD8^{+} T cells, the percentage and number of (C-E) regulatory T cells, the percentage of (F, G) CD11c^{+} dendritic cells, and (H, I) CD19^{+} B cells. (A, C, F, H) Representative flow cytometric profiles, and (B, D, E, G, I) statistical analyses are shown. The data are expressed as mean ± SD from one of three independent experiments with similar results (4-8 mice per group in each experiment). *$p < 0.05$ versus control cell group.

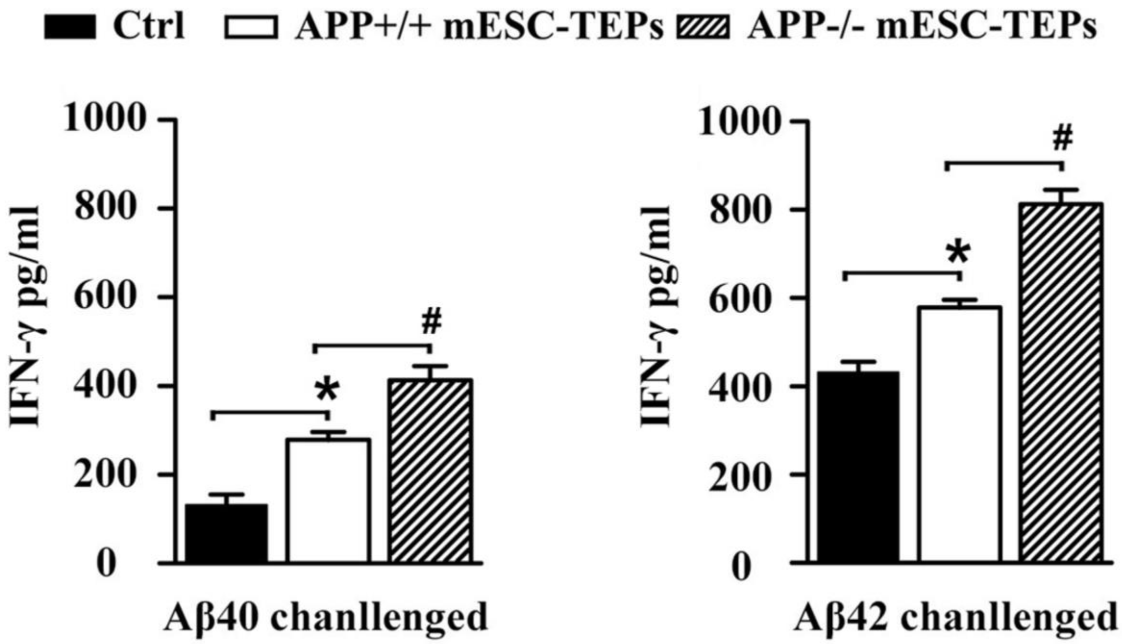

Supplemental Figure 5. Transplantation of $APP^{+/+}$ or $APP^{-/-}$ mESC-TEPs leads to increased production of IFNγ from splenocytes. 3XTg-AD mice (12-month-old) were injected i.t. with $APP^{-/-}$ mESC-TEPs, $APP^{+/+}$ mESC-TEPs, or control cells as in Figure 2. Two and a half months later, the splenocytes (normalized to 1 X 10^5 T cells/well) were cultured with Aβ40 or Aβ42 in the presence of anti-CD3 antibody for 3 days. The supernatant was analyzed for the content of IFNγ by ELISA. The data are expressed as mean ± SD from one of three independent experiments with similar results (4-8 mice per group in each experiment). $^{*}p < 0.05$ versus control cell group, $^{\#}p < 0.05$ versus $APP^{+/+}$ mESC-TEP group.

Supplemental Table 1. Primers for RT-PCR and qRT-PCR

Gene		Sequence 5'-3'
	Forward	ATGCAGCGAGAAGAGCACTAA
APP	Reverse	CGGCCAACGGGCAGC
	Forward	AGGTCGGTGTGAACGGATTTG
GAPDH	Reverse	TGTAGACCATGTAGTTGAGGTCA
	Forward	AGCATACAGGTCCTGGCATCTTGT
ppia	Reverse	CAAAGACCACATGCTTGCCATCCA
	Forward	ACAGCAAGGCGAAAAAGGATG
infg	Reverse	TGGTGGACCACTCGGATGA
	Forward	AGATCACATTCACGGTGCTGGCTA
icam1	Reverse	AGCTTTGGGATGGTAGCTGGAAG
	Forward	TGTGAAGGGATTAACGAGGCTGGA
vcam1	Reverse	CCATGTTTCGGGCACATTTCCACA
	Forward	CATCCACGTGTTGGCTCA
ccl2	Reverse	GATCATCTTGCTGGTGAATGAGT
	Forward	AACTGCATCCATATCGATGAC
cxcl10	Reverse	GTGGCAATGATCTCAACAC
	Forward	GTTACCTCAGTTCATCCA
Ccr2	Reverse	CAAGGCTCACCATCATCGTAGTC
	Forward	TTGGCTTCCCTGGAGGTCGAG
SRA1	Reverse	ACACAGGAACCAATGTCATTTG
